# Flexible Vibration Sensors with Omnidirectional Sensing Enabled by Femtosecond Laser-Assisted Fabrication

**DOI:** 10.3390/polym17020211

**Published:** 2025-01-16

**Authors:** Yaojia Mou, Cong Wang, Shilei Liu, Linpeng Liu, Ji’an Duan

**Affiliations:** 1 State Key Laboratory of Precision Manufacturing for Extreme Service Performance, College of Mechanical and Electrical Engineering, Central South University, Changsha 410083, China; 233712156@csu.edu.cn (Y.M.); wangcong@csu.edu.cn (C.W.); duanjian@csu.edu.cn (J.D.); 2 Hunan Agricultural Forestal and Industrial Prospective Design Institute, Co., Ltd., Changsha 410083, China; liushilei0610@foxmail.com; 3 Department of Mechanical Engineering, The Hong Kong Polytechnic University, Hong Kong 999077, China

**Keywords:** vibration, sensors, femtosecond laser, low cost, easy to manufacture

## Abstract

Vibration sensors are integral to a multitude of engineering applications, yet the development of low-cost, easily assembled devices remains a formidable challenge. This study presents a highly sensitive flexible vibration sensor, based on the piezoresistive effect, tailored for the detection of high-dynamic-range vibrations and accelerations. The sensor’s design incorporates a polylactic acid (PLA) housing with cavities and spherical recesses, a polydimethylsiloxane (PDMS) membrane, and electrodes that are positioned above. Employing femtosecond laser ablation and template transfer techniques, a parallel groove array is created within the flexible polymer sensing layer. This includes conductive pathways, and integrates stainless-steel balls as oscillators to further amplify the sensor’s sensitivity. The sensor’s performance is evaluated over a frequency range of 50 Hz to 400 Hz for vibrations and from 1 g to 5 g for accelerations, exhibiting a linear correlation coefficient of 0.92 between the sensor’s voltage output and acceleration. It demonstrates stable and accurate responses to vibration signals from devices such as drills and mobile phone ringtones, as well as robust responsiveness to omnidirectional and long-distance vibrations. The sensor’s simplicity in microstructure fabrication, ease of assembly, and low cost render it highly promising for applications in engineering machinery with rotating or vibrating components.

## 1. Introduction

With the rapid expansion of smart devices and the Internet of Things (IoT), the degree of automation across various manufacturing industries has seen significant advancements. As a result, the real-time monitoring and predictive maintenance of equipment have emerged as critical research areas [1,2,3,4,5]. During the operation of machinery, mechanical vibrations are invariably generated, and their analysis via sensors can elucidate characteristics such as frequency, amplitude, velocity, and acceleration [4,6,7,8,9,10]. This facilitates the continuous monitoring of equipment performance, enabling optimization and the early diagnosis of potential failures, thus triggering proactive alerts [11,12,13]. As a result, vibration sensors hold immense developmental potential. Among these, flexible sensors have gained prominence due to their wide applications and growth prospects. These sensors offer notable advantages, including exceptional flexibility, lightweight portability, and adaptability to complex surfaces, while maintaining high sensitivity and accuracy, making them superior in scenarios where traditional rigid sensors may struggle [14,15,16,17,18,19].

Currently, laser interferometric and strain gauge sensors dominate engineering applications [20,21,22,23,24,25,26,27,28,29]. For instance, Huang et al. [4] designed a 6H-SiC sapphire fiber optic vibration sensor capable of withstanding high temperatures. This sensor utilizes sapphire fibers to convert vibration signals into optical signals via a laser input into the sensor’s resonant cavity, with parameters obtained through optical demodulation. Integrated with a cantilever structure, it achieves a testing sensitivity of 0.9997 Hz/Hz and full-scale accuracy of 0.44%, even at 1200 °C. However, both types of sensor mentioned above have notable limitations. Laser interferometric sensors typically consist of multiple precision optical components, such as a laser source, beam splitters, mirrors, and interferometers. These components require extremely high installation and alignment precision to ensure optical path stability; any misalignment can introduce additional strain measurement errors. Moreover, maintaining the requisite optical path conditions for interference complicates the sensor’s architecture, resulting in increased design and manufacturing costs [13,30,31,32,33,34,35]. On the other hand, the complexity of strain gauge vibration sensors lies primarily in the design of their sensitive elements, the high degree of integration with mechanical structures, the intricate bridge circuit systems, and the demands for signal amplification and processing. These sensors must also be securely attached to the surface of mechanical structures to detect minute vibrations and deformations, further complicating their overall design and manufacturing. Additionally, due to their high sensitivity to environmental factors, advanced packaging design is crucial for strain gauge sensors, significantly raising both design and production costs [36,37,38,39,40,41,42,43]. Therefore, the development of vibration sensors capable of detecting a wide frequency range and high degree of acceleration, and which are also structurally simple and easy to assemble, remains a challenging task—particularly in terms of enhancing mechanical monitoring precision and expanding practical applications.

To address these challenges, this paper presents a high-sensitivity flexible vibration sensor, based on a piezoresistive effect, for monitoring high dynamic vibrations and accelerations. The vibration sensor comprises PLA housing with cavities and a spherical pit, an Ag/PDMS/MWCNTs/CBs-sensitive membrane, and surface electrodes. Utilizing femtosecond laser ablation and template transfer techniques, a meticulously crafted parallel groove array with conductive pathways is integrated into the flexible polymer sensing layer. This innovative design facilitates the transformation of mechanical deformation into discernible resistance changes, thereby enabling the sensor to generate electrical signals in response to vibrational stimuli [44,45]. Moreover, the sensor features a simple structural design and assembly process, wherein the central part of the vibration-sensitive membrane is suspended and the outer edges are fixed. This configuration ensures that the membrane vibrates and deforms in response to vibration signals, thereby enhancing its response capability [46,47]. Furthermore, a stainless-steel ball, constrained to move along the z-axis, is incorporated into the sensor cavity as a resonator. Experimental results demonstrate that the vibration frequency detection range of the sensor spans from 50 Hz to 400 Hz, while the acceleration detection range is from 1 g to 5 g. The linear correlation coefficient between the vibration amplitude and the output signal across various accelerations soars as high as 0.92. The sensor exhibits excellent sensitivity and stability in practical applications, such as monitoring the vibrations of equipment like electric drills and mobile phones. The sensor’s response capability is further evaluated by controlling the orientation and distance of vibration source signals, indicating its substantial potential for mechanical equipment vibration detection applications.

## 2. Materials and Methods

### 2.1. Materials

Materials: PDMS (Sylgard 184) was bought from Dow Corning Co., Ltd., Midland, MI, USA. MWCNTs (average diameter 10–20 nm, average length 10–30 µm) were purchased from Nanjing XFNANO Materials Tech Co., Ltd., Nanjing, China. Ethyl acetate was purchased from Shanghai Sinopharm Chemical Reagent Co., Ltd., Shanghai, China. CBs (ECP-600JD) were purchased from Tianjin Aiweixin Chemical Technology Co., Ltd., Tianjin, China. Epoxy resin was obtained was purchased from Ausbond Co., Ltd., Shenzhen, China. Silver paste (JY12) was purchased from Shanghai Julong Electronic Technology Co., Ltd., Shanghai, China. Copper paste was bought from Dongguan Hengchuang Adhesive Products Co., Ltd., Dongguan, China.

### 2.2. Preparation of Conductive Ink

First, the components reagent A and reagent B were mixed in a 10:1 ratio to prepare the PDMS prepolymer. Then, multi-walled carbon nanotubes (MWCNTs), carbon black (CB), PDMS prepolymer, and ethyl acetate were added in a weight ratio of 1:5:30:500, respectively. The mixture was magnetically stirred at 500 rpm for 30 min to ensure thorough mixing. Finally, the uniformly blended solution was subjected to ultrasonic treatment for 5 min to achieve optimal dispersion. The conductive ink was then prepared.

### 2.3. Fabrication of Vibration Sensor

The sensor sample was prepared through the following steps: First, a femtosecond laser was used to ablate a groove array onto a metal sheet, which served as a fabrication mold. This mold was then thoroughly cleaned with anhydrous ethanol to remove the oxides generated during the high-temperature ablation process. Next, epoxy resin and hardener were mixed in a weight ratio of 2:1 and stirred until a homogeneous mixture free of strands was obtained. Then, the mixture was poured into the metal mold to replicate the microstructure of the sensitive layer. After curing for 60 min, the microstructural mold was complete. Subsequently, PDMS prepolymer and hardener were mixed in a weight ratio of 10:1, and we performed thorough stirring and vacuum degassing before pouring the mixture into the epoxy mold. The assembly was placed in a constant-temperature chamber set to 70 °C for 60 min, resulting in a PDMS film with a groove array on its surface, which functioned as the sensitive layer of the sensor. Afterward, pre-prepared conductive ink was uniformly sprayed onto the conductive path areas of the PDMS film; the film was then dried in a 100 °C constant-temperature chamber for 1 h to ensure the complete evaporation of ethyl acetate and the full curing of the PDMS layer. Finally, silver paste was applied to both ends of the conductive path on the film and dried at 100 °C for 1 h. Conductive copper foil was then attached, and leads were soldered. The sensitive layer was subsequently bonded to the sensor housing using specialized adhesive and surface treatment agents, thus completing the sensor sample preparation.

**Figure 1:**
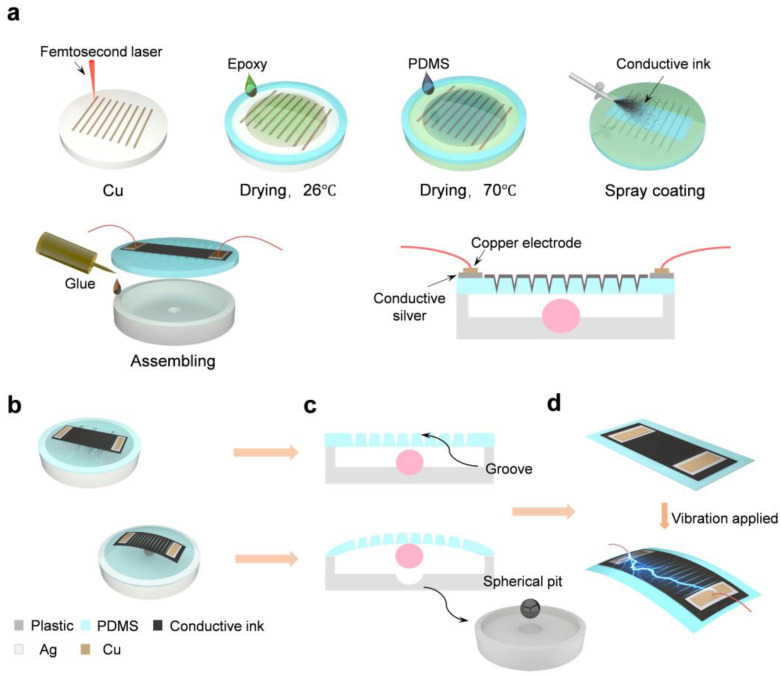
An overall model of the sensor and its fabrication process. (**a**) The fabrication process of the sensor. (**b**) A 3D model of the sensor and its state after being subjected to vibration. (**c**) A 2D model of the sensor and its state after being subjected to vibration. (**d**) A 3D model of the sensor’s working mechanism.

Figure 1a details the complete fabrication process of the sensor. First, a femtosecond laser is used to ablate a metal sheet, yielding nine groove arrays. Each of them has a length of 8 mm and the interval between them is 0.5 mm. Then, a prepared liquid epoxy resin is cast onto the metal template and cured at room temperature for one hour to form a sacrificial mold that replicates the groove arrays on the metal mold. The prepared PDMS mixture is poured into the mold and cured for one hour in a constant-temperature oven at 70 °C. After peeling it from the mold, an 8 mm radius PDMS film with surface groove arrays is obtained. Due to the excellent flexibility and surface properties of PDMS, the PDMS film is selected as the base material for the sensor’s sensitive layer.

### 2.4. Design of the Groove-Based Composite Flexible Sensor

Figure 1b shows the architecture of the sensor, which is predominantly constituted by PLA housing with cavities and a spherical pit, copper electrodes, a stainless-steel ball, and a sensitive membrane made of Ag/PDMS/MWCNTs/CB. When the sensor is stimulated by mechanical vibration signals, the 200 μm thick PDMS film oscillates under the influence of inertia, causing the width of the groove on its surface to change. This alters the sensor’s resistance, thereby converting mechanical deformation into electrical signals. Additionally, a stainless-steel ball with a diameter of 2 mm inside the sensor’s cavity bounces up and down, further increasing the deformation of the sensitive membrane and surface groove array, effectively improving the sensor’s sensitivity. Furthermore, as shown in Figure 1c, the bottom of the housing cavity is designed with a spherical pit that matches the size of the stainless-steel ball, effectively confining the ball’s movement along the x- and y-axes. This design ensures that the ball can only bounce vertically in response to vibration signals, reducing the input of interference strain and preventing uneven stress transfer and additional energy loss caused by friction between the ball, the cavity bottom, and the vibration-sensitive membrane. This refinement substantially bolsters the sensor’s response linearity.

Upon the completion of the aforementioned tasks, a conductive pathway region with a length of 12 mm and a width of 5 mm is formed on the PDMS film using a stencil. Next, the previously prepared conductive ink is sprayed evenly over this region with a spray gun, ensuring that the groove arrays fully penetrate the conductive path, thus limiting the electron transfer path during electrical conduction. This ensures the effectiveness of the groove microstructures for the sensor and the stability of its operation. Finally, the housing and the sensor’s sensitive layer are assembled using specialized glue, and copper electrodes and wires are added to both ends of the conductive path. The fabrication of the flexible sensor is then completed.

Then, Figure 1d illustrates the sensor’s dynamic response under vibration signal stimulation. When a vibration signal is applied, the sensor oscillates vertically due to its inherent material properties. The vertical rebound of the internal stainless-steel resonator further amplifies this motion, resulting in the deformation of the sensitive layer. Consequently, variable groove widths are generated within the surface array of the sensitive layer. These fluctuations lead to changes in the sensor’s resistance, enabling the efficient transduction of mechanical vibration signals into electrical output.

### 2.5. Characteristics of the Vibration Sensor

The microstructural array of groove on the surface of the sensor’s flexible film was characterized using an optical microscope (Nikon, Tokyo, Japan). A vibration excitation system, consisting of a vibration exciter (SA-JZ002, ShiaoTech Co., Ltd., Wuxi, China), a power amplifier (SP-PA003, ShiaoTech Co., Ltd., Wuxi, China), a signal generator (DG1022Z, RIGOL, Beijing, China), a dynamic signal analyzer (SA1808A2, ShiaoTech Co., Ltd., Wuxi, China), and a commercial accelerometer (SACL001ZKE, ShiaoTech Co., Ltd., Wuxi, China), was used in subsequent experiments to generate vibration signals at different frequencies. The resistance signals of the sensor were collected using a digital multimeter (DAQ6510, KEITHLEY, Cleveland, OH, USA). The acceleration of the vibration signal and the voltage signal at the sensor terminals were acquired using a dynamic data acquisition system (SA1808A2, ShiaoTech Co., Ltd., Wuxi, China).

## 3. Results and Discussion

### 3.1. Performance of the Groove-Based Composite Flexible Sensor

**Figure 2:**
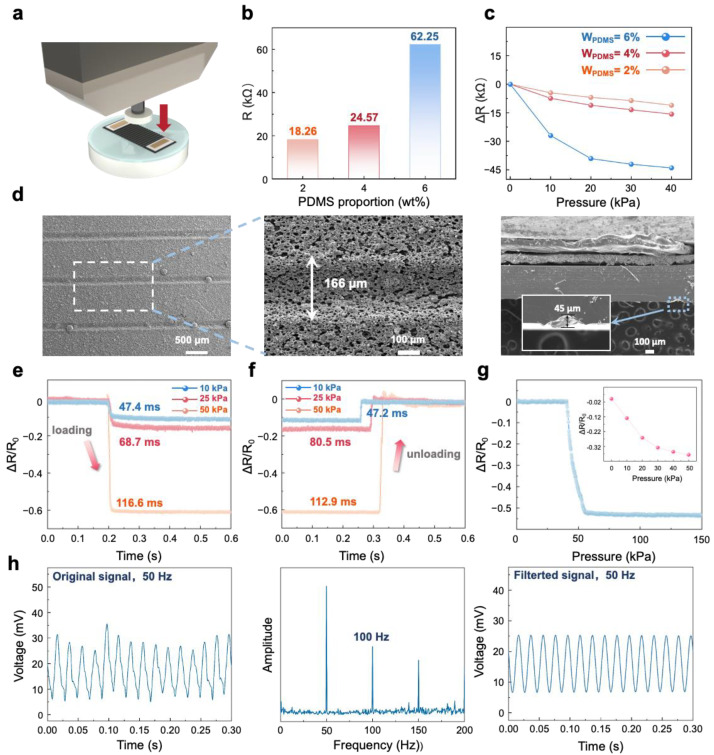
The conductive ink proportion comparison experiment, the sensor working mechanism, and SEM images, as well as experimental flowcharts and result graphs. (**a**) An image of the comparative experimental setup. (**b**) The initial resistance of different PDMS proportions. (**c**) The results of the control experiment. (**d**) An SEM image illustrating the sensor’s operational mechanism. (**e**) Response time of the sensor at pressures of 10 kPa, 25 kPa and 500 kPa, respectively. (**f**) The recovery time of the sensor at pressures of 10 kPa, 25 kPa and 500 kPa, respectively. (**g**) Relative resistance change curve of the pressure sensor at pressures from 0 to 150 kPa. (**h**) The results of the experiment.

Before conducting the performance tests of the sensor, preliminary experiments were performed on the conductive ink. The mass fractions of PDMS in the conductive ink were varied to 2%, 4%, and 6%, while we kept the other components of the ink constant. These three formulations were then subjected to comparative testing. In the experiments, a force gauge was used to apply varying pressures to the sensor’s sensitive layer, and the resulting resistance changes were recorded. The experimental setup and results are presented in Figure 2a–c. Figure 2b displays the initial resistance of the conductive inks with the three different PDMS proportions. Figure 2c illustrates that the conductive ink with 6% PDMS exhibits a significantly higher resistance change under applied pressure compared to the other two formulations, indicating that the selected ink composition ensures the sensor achieves optimal sensitivity.

After conducting preliminary tests on the conductive ink, the optimal formulation was selected for fabricating the sensing layer of the sensor. Figure 2d presents an SEM image of the surface of the sensor’s sensitive layer, revealing uniformly distributed grooves, approximately 166 µm in width, on the PDMS layer. These grooves are evenly coated with conductive ink, enhancing the sensor’s conductivity and significantly improving its sensitivity. Subsequently, the performance of the fabricated sensitive layer film was evaluated through relevant testing. Using a stepper motor and a force gauge, the response characteristics and response time of the composite material layer were tested under different pressures. As shown in Figure 2e,f, under the applied pressures of 50 kPa, 25 kPa, and 10 kPa, the measured response times were 116.6 ms, 68.7 ms, and 47.4 ms, respectively, while the corresponding recovery times were 112.9 ms, 80.5 ms, and 47.2 ms. These results indicate that the sensor’s sensitive layer demonstrates excellent response speed at various pressure levels, fully confirming its superior dynamic performance.

Additionally, we tested the sensor’s response behavior under given pressure conditions, and the results are shown in Figure 2g. The response curve reveals that within the 0–50 kPa pressure range, the sensor exhibits higher sensitivity, with the resistance decreasing rapidly as pressure increases. However, within the 50–150 kPa range, the sensitivity decreases, and the resistance change becomes more gradual. This phenomenon aligns closely with our theoretical analysis of the sensor’s working mechanism, further validating the rationality of the design.

With the fundamental characteristics and operational mechanism of the sensor clearly established, we proceeded to perform performance testing, with a specific focus on frequency response. A performance testing system was constructed as follows: The test setup involved securely affixing the sensor to a flat plate connected to the output shaft of the vibration exciter, thereby eliminating any extraneous stress or deformation that could potentially interfere with the results. The testing system comprised an arbitrary waveform generator, a power amplifier, an exciter, a data acquisition device, and analysis equipment. Vibration signals of various waveforms and frequencies are generated by the waveform generator, amplified by the power amplifier, and then applied to the sensor via the exciter, ensuring the synchronous vertical vibration of the sensor prototype with the exciter. A commercial accelerometer is also mounted onto the output shaft to quantify the exciter’s specific amplitude output.

In light of the data acquisition system’s capacity to only record voltage signals, the piezoresistive sensor is integrated in series with a fixed 2.3 kΩ resistor (R1) within a 5 V circuit. This arrangement facilitates the transformation of vibration-induced resistance variations into voltage signals that can be accurately captured and analyzed. The results, shown in Figure 2h, include three plots: the sensor’s voltage response to a 50 Hz vibration signal at a controlled acceleration of 1 g (achieved via the power amplifier), the FFT analysis of this response, and the filtered voltage response after the removal of ambient interference signals. The data indicate that the sensor’s response to the 50 Hz vibration signal is both accurate and stable.

**Figure 3:**
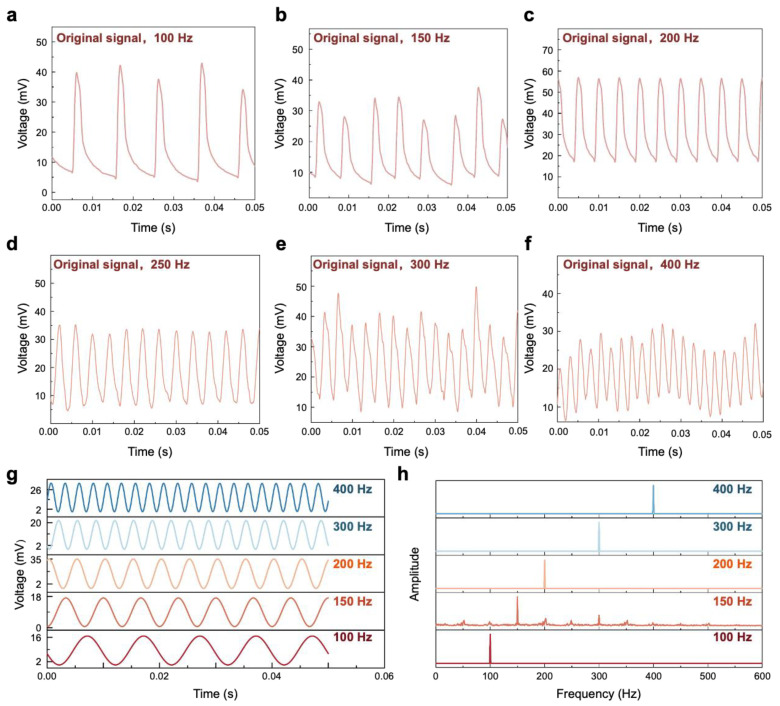
Sensor responses to different vibration frequencies. (**a**) The response of the sensor to an input vibration frequency of 100 Hz. (**b**) The response of the sensor to an input vibration frequency of 150 Hz. (**c**) The response of the sensor to an input vibration frequency of 200 Hz. (**d**) The response of the sensor to an input vibration frequency of 250 Hz. (**e**) The response of the sensor to an input vibration frequency of 300 Hz. (**f**) The response of the sensor to an input vibration frequency of 400 Hz. (**g**) The FFT analysis of the sensor’s response to input vibration signals ranging from 100 to 400 Hz. (**h**) The results of the sensor’s response after filtering for input vibration signals in the 100–400 Hz range.

Figure 3a–f present a comprehensive analysis of the sensor’s voltage response at output vibration frequencies of 100, 150, 200, 300, and 400 Hz. It is evident that the sensor demonstrates a highly regular and stable response to the applied vibration signals. As demonstrated in these figures, the sensor’s peak periodic responses are in exact correspondence with the periods of the input signals, underscoring its precise tracking capability. Figure 3g shows the FFT analysis of the sensor’s response to input vibration signals ranging from 100 to 400 Hz. Prominent peak signals are observed at frequencies that match each input frequency, indicating a robust correlation with the exciter’s output frequency. Figure 3h, which depicts the response after filtering out environmental interference, also confirms the sensor’s accuracy and stability when exposed to vibration signals in the 100–400 Hz range.

**Figure 4:**
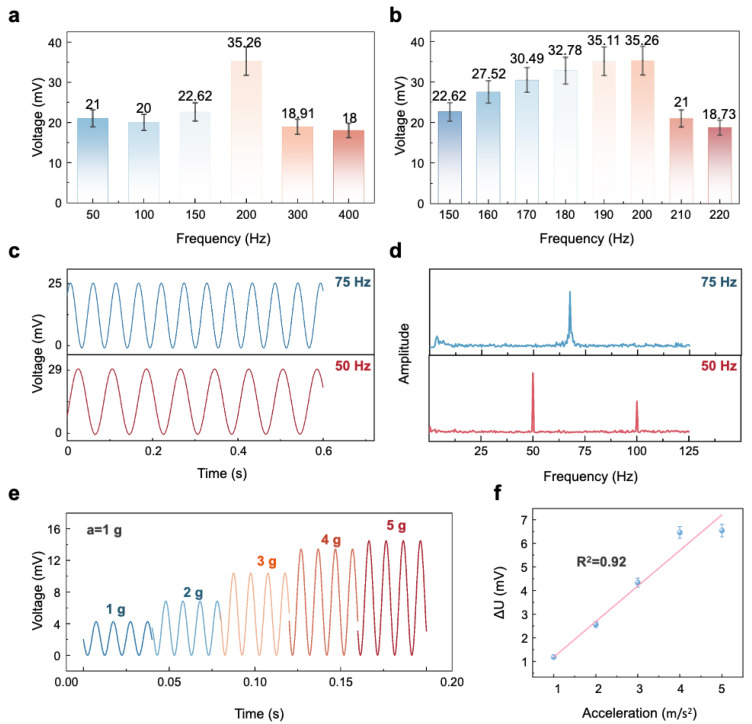
Detailed experiments on the sensor’s response to resonance and its performance under low-frequency conditions. (**a**) The peak response of the sensor to vibrations at frequencies ranging from 50 Hz to 300 Hz. (**b**) Peak values of the sensor’s response signals for vibrations at frequencies between 150 Hz and 220 Hz (an increment of 10 Hz). (**c**) The FFT analysis of the sensor’s response to input vibration signals at 50 Hz and 75 Hz. (**d**) The results of filtering the sensor’s response to input vibration signals at 50 Hz and 75 Hz. (**e**) The response of the sensor to mechanical vibrations at a constant frequency of 100 Hz but with varying accelerations. (**f**) The linearity of the sensor’s response to different acceleration signals.

In Figure 3h, it can be observed that the maximum output voltage amplitude diminishes with increasing vibration frequency, except for a notable exception at 200 Hz. This aligns with the low-frequency enhancement effect. The anomalously high peak observed at 200 Hz (Figure 4a) suggests possible resonance phenomenon. To probe this hypothesis further, an in-depth analysis of the vibration signals within the 150–220 Hz band is undertaken. As shown in Figure 4b, the maximum output voltage amplitude reaches its peak at 200 Hz and then gradually decreases, indicating that the resonance frequency is approximately 200 Hz.

The aforementioned article provides detailed experimental analysis of the sensor’s response to high-frequency signals; however, it is equally essential to evaluate its response to low-frequency signals, which are typically associated with structural vibrations and the failures of large machinery. Subsequently, this research is extended to include an evaluation of the sensor’s performance with low-frequency vibration signals beneath 100 Hz. As shown in Figure 4c,d, the sensor demonstrates stability, uniformity, and accuracy in responding to signals at 75 and 50 Hz. Furthermore, the FFT analysis presented in Figure 4d indicates a robust correlation between the sensor’s response and the output of these low-frequency signals.

Subsequently, by adjusting the power amplifier, we examined the sensor’s response to signals at 100 Hz with varying vibration accelerations. It is evident that the maximum relative voltage variation increases with the applied acceleration, as depicted in Figure 4e. Further analysis involved a linear fitting of the data, presented in Figure 4f, yielding a linear correlation coefficient of 0.92. This finding indicates that the designed vibration sensor exhibits commendable linearity, effectively addressing the challenge faced by some flexible sensors in balancing sensitivity and linearity.

### 3.2. Application of the Groove-Based Composite Flexible Sensor

**Figure 5:**
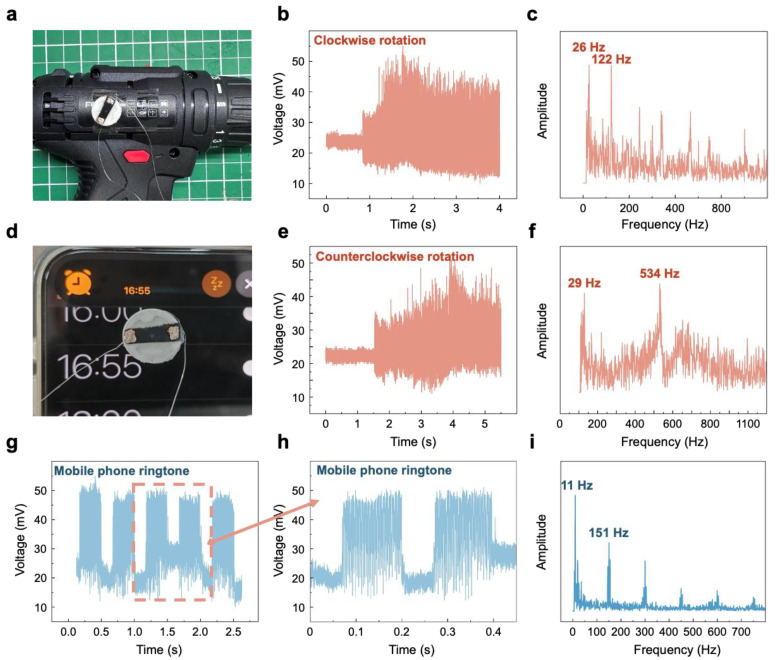
Applications of the sensor on a power drill and mobile phone (**a**). A photograph of the sensor applied to the power drill. (**b**) The voltage response of the sensor to the vibration signals during the forward rotation of the drill. (**c**) The FFT analysis of the voltage response signals from the sensor during the forward rotation of the drill. (**d**) A photograph of the sensor applied to the mobile phone. (**e**) The voltage response of the sensor to the vibration signals during the reverse rotation of the drill. (**f**) The FFT analysis of the voltage response signals from the sensor during the reverse rotation of the drill. (**g**) The voltage response of the sensor to the vibration signals from the mobile phone. (**h**) An amplified view of the sensor’s voltage response to the mobile phone’s vibration signals. (**i**) The FFT analysis of the voltage response signals from the sensor regarding the mobile phone’s vibration signals.

Then, to demonstrate the potential of the designed and fabricated sensor in monitoring mechanical operating states indicative of vibrations, we integrated the sensor into tangible vibration-monitoring scenarios. As shown in Figure 5a, the sensor was installed onto a power drill, which operated in both clockwise and counterclockwise modes. Figure 5b,e attest that, irrespective of the operational mode, the sensor evinced a pronounced response upon the drill’s activation. It transitioned from a steady initial linear signal to one that mirrored the vibration signals emitted by the drill. Further FFT analysis of the output electrical signals reveals the characteristic frequencies of the drill during operation at approximately 26 Hz, 122 Hz, 29 Hz, and 534 Hz, as depicted in Figure 5c,f. Additionally, we affixed the sensor to a mobile phone to assess its response to vibration signals when the ringtone was activated, as shown in Figure 5d. Figure 5g,h indicate that the sensor’s response to the mobile phone’s vibration signals was also consistent and stable, aligning with the signal period. Following FFT analysis, the characteristic frequency of the mobile phone’s ringtone was ascertained to be approximately 11 Hz.

**Figure 6:**
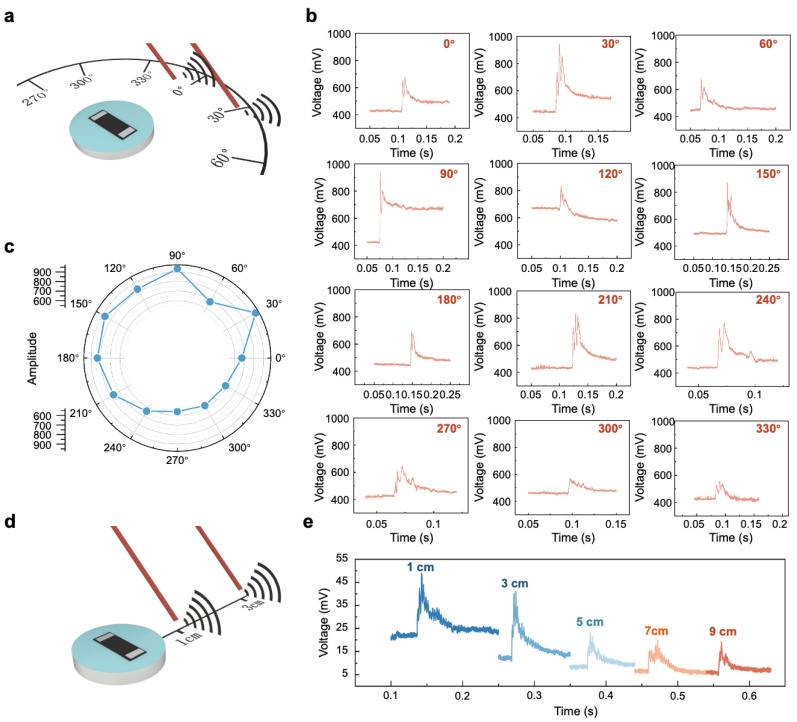
The evaluation of the sensor’s omnidirectional and long-distance response capabilities. (**a**) A schematic representation of the omnidirectional response capability testing experiment. (**b**) The specific response of the sensor to impact signals at various angles. (**c**) The comprehensive response of the sensor to impact signals at different angles. (**d**) The schematic representation of the long-distance response capability testing experiment. (**e**) The results of the sensor’s response to impact signals at varying distances.

To rigorously evaluate the sensor’s performance, it was meticulously placed at a stationary location, and uniform impact signals of consistent magnitude were systematically delivered around it at increments of 30°, as demonstrated in Figure 6a. This test is designed to evaluate the sensor’s responsiveness at various angles, thereby examining its omnidirectional vibration response capabilities. The experimental outcomes, as detailed in Figure 6b,c, disclose a uniform response from the sensor in all directional tests, highlighting its robust performance, irrespective of orientation. Similarly, to evaluate the influence of distance on the sensor’s responsiveness, impact signals of equal intensity are applied at varying distances along a straight line, as shown in Figure 6d. The findings presented in Figure 6e indicate that the sensor accurately responded to signals across all measured distances, with the voltage signal gradually diminishing as the distance increased. This result substantiates the sensor’s competence in the accurate detection of vibrations at considerable distances.

## 4. Conclusions

In conclusion, this study introduces a highly sensitive flexible vibration sensor that capitalizes on the piezoresistive effect and which is specifically designed for monitoring dynamic vibrations and accelerations. The sensor is constructed with a PLA casing featuring a cavity, a PDMS membrane, a sensitive functional membrane, and surface electrodes. Experimental findings demonstrate the sensor’s capability to accurately detect vibration signals across a broad frequency spectrum from 50 Hz to 400 Hz, and to measure accelerations varying from 1 g to 5 g, achieving a commendable linear correlation coefficient of 0.92 between the voltage signal and acceleration. The sensor demonstrates stable and accurate responses to vibration signals from various vibration sources, including electric drills and mobile phone ringtones, while also exhibiting excellent responsiveness to omnidirectional and long-distance vibrations. The simplicity of the sensor’s microstructural design, the ease with which it can be assembled, and its cost-effectiveness highlight its significant potential for use in engineering machinery with rotating or vibrating components.

## Data Availability

The original contributions presented in the study are included in the article, further inquiries can be directed to the corresponding author.
